# An itchy rash and muscle weakness

**DOI:** 10.1002/ccr3.4750

**Published:** 2021-08-30

**Authors:** Brian L. Risavi, Justin Puller, Kristin Juhasz

**Affiliations:** ^1^ Lake Erie College of Osteopathic Medicine Erie Pennsylvania USA; ^2^ UPMC Hamot Erie Pennsylvania USA

**Keywords:** dermatomyositis, muscle weakness, myopathy, rash

## Abstract

Dermatomyositis is a rare disease affecting primarily skin and muscles and is associated with malignancies, especially in at‐risk patients. Clinical presentations vary widely but proximal muscle weakness and typical skin findings should prompt consideration of the diagnosis. Immunosuppressive therapy is required, as is continued screening for malignant disease during follow‐up.

## Graphical abstract

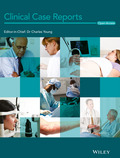

## 1 INTRODUCTION

Dermatomyositis is a rare disease affecting primarily skin and muscles and is associated with malignancies, especially in at‐risk patients. Clinical presentations vary widely but proximal muscle weakness and typical skin findings should prompt consideration of the diagnosis. Immunosuppressive therapy is required, as is continued screening for malignant disease during follow‐up.

Dermatomyositis is a rare idiopathic inflammatory myopathy characterized by a complement‐mediated destruction of capillaries with ischemic muscle changes.^1^, ^2^ This is a disorder affecting primarily skin and muscle.^3^ It is associated with internal malignancy, including nasopharyngeal, lung, ovarian, colorectal, and others.

Skin manifestations include an erythematous heliotrope rash of the periorbital area (upper eyelid), Gottron's papules (faint red or violet papules occurring over the knuckles), Gottron's sign (rash over the palmar aspect of the hand), and facial erythema (malar area, forehead, temples, medial angle of eyes) with sparing of the nasolabial fold.^1^ A “V” neck sign, redness of the upper chest, and “shawl” sign (symmetrical violaceous erythema extending from the nape of the neck to the posterior aspect of the shoulders to upper back) are also hallmarks. Fissuring, hyperkeratotic, and scaly hands are referred to as “mechanic's hands”.^3^ Periungual telangiectasias may also be seen. Scalp rash is frequently overlooked or misdiagnosed and is typically very pruritic with atrophic, erythematous, and scaly plaques.^4^ Characteristic muscular manifestations include proximal weakness and difficulty rising from a sitting or supine position.^1^

CPK levels are often elevated. EMG (electromyogram) is used to assess myopathy and may reveal spontaneous activity, small amplitudes, and short potentials. Nerve conduction is normal, compatible with diffuse muscle disease. Treatment should be initiated in the ED if suspicion is high and includes systemic corticosteroids initially. Intravenous immunoglobulin can be used for severe cases requiring admission or if high‐dose steroids are contraindicated.^5^

## 2 CASE

A 62‐year‐old Caucasian female with a history of COPD and one pack per day smoker for 50 years presents complaining of 4–5 weeks of diffuse progressive weakness and myalgias of the shoulders and thighs without focal deficits. She reported difficulty getting out of a chair and difficulty walking. She further reported difficulty taking a drink from a cup of tea, which she had to hold with both hands secondary to muscle weakness. She also reported a very itchy rash of her scalp, back of her hands, and upper chest/back, and “cracked” skin of her hands. She denied any dysphagia. Review of systems was otherwise negative. She was initially evaluated by her primary care physician over 4 weeks and topical antifungals were prescribed without improvement. She then presented to the emergency department for further evaluation.

Examination revealed an erythematous scaly rash of the scalp, left forehead, left neck, and upper chest/back. Gottron's papules were noted along with cracked skin of her digits. Weakness of the shoulders bilaterally (4/5) and thighs (3/5) was noted. There were no cranial nerve deficits. No focal neurologic deficits were noted. Neurology and rheumatology services were consulted and evaluated the patient.

Diagnostic work‐up revealed the following: CPK 353 (24–170), ESR 33 (0–30); TSH, RF, dsDNA, Sjogren AB, cardiolipin IgM, lupus anticoagulant, all were negative. Aldolase was 6.0 (0–8.1). Jo‐1 antibody was negative. PL‐12, MI‐2, KU, OJ, SRP, and nJO‐1 were all negative as well. Vastus medialis biopsy revealed diffuse perivascular chronic inflammation with evidence of inflammation and/or necrosis. EMG revealed electrodiagnostic evidence for a moderate myopathy affecting proximal muscles. An MRI of the cervical spine was done to assist in differentiating neuropathologic disease which demonstrated no acute pathology. Evaluation for associated malignancies included negative CTs of the chest/abdomen/pelvis and mammogram. Outpatient colonoscopy was scheduled.

This patient was admitted to the hospital for further evaluation and responded well to corticosteroids and methotrexate and had an uneventful hospital course. She was discharged home on a 30‐day course of corticosteroids and methotrexate with rheumatology and neurology follow‐up. Follow‐up pulmonary function testing revealed a moderate airway obstructive pattern. Muscle weakness increased with proximal strength 2–3/5 and distal strength 4/5. To summarize the patient's timeline of pathology, the patient developed shortness of breath and hemoptysis 22 months after the emergency department visit. A chest CT identified a right lung mass, determined to be non‐small cell carcinoma by biopsy. Chemotherapy and radiation treatment were started and 11 months thereafter the patient passed.

## 3 DISCUSSION

Dermatomyositis is a rare idiopathic inflammatory myopathy with a prevalence of 5–22 per 100,000 as well as a broad range of clinical presentations, making diagnosis difficult.^6^ It is characterized by a complement‐mediated destruction of the capillaries and small arterioles, resulting in microinfarctions of the muscle. Peak incidence in adults occurs between age 40–50, although it may occur at any age, including children.^7^, ^8^ Females are affected nearly twice as often as males. The etiology is thought to be multifactorial, with infection, drugs, and lifestyle factors likely playing a role.^9^ Evidence suggests abnormalities involving the interferon pathway as well.^10^

The case presented offers an example of a rather typical presentation with several common findings. The patient was evaluated twice in the outpatient setting and did not respond to conservative treatment. She subsequently presented to the emergency department for further evaluation. Given the rarity of this disease and similarity to other musculoskeletal pathology, the diagnosis is often challenging.^7^ Patients may present to the ED for new or worsening symptoms as in this case, or simply for a second opinion.

The most common manifestations involve the skin and muscles.^1^ Cutaneous findings include Gottron's papules (pink‐violaceous papules on the dorsal aspect of the hands, particularly over the metacarpophalangeal and interphalangeal joints), Gottron's sign (pink‐violaceous erythema over the joints), facial erythema of the malar, forehead, and temple regions, and a heliotrope rash of the periorbital area.^3^ Hyperkeratotic, cracked skin of the hands, typically the index and middle fingers, is referred to as “mechanic's hands”.^3^ Poikiloderma of exposed skin often demonstrates the shawl sign on the upper back and the V sign on the upper neck (pink‐violaceous erythema of these areas).^3^ Psoriaform changes in the scalp are frequently diffuse, scaly, and pruritic.^4^ Some cases demonstrate periungual erythema of the nail folds, and midfacial erythema of the nasolabial folds, and pink‐violaceous scaly erythema of the lateral thighs referred to as the “holster sign”.^10^ Calcinosis of the subcutaneous tissues is a rare occurrence that has been reported at any age but is more common in pediatric patients.^5^, ^11^ Muscle involvement is typically proximal and symmetric. Patients often complain of difficulty getting out of a chair or ambulating up steps. They may also complain of trouble speaking or swallowing.^12^

To establish the diagnosis, other causes of muscle weakness must first be excluded which will often require EMG and nerve conduction studies. Recently, new diagnostic criteria (European League Against Rheumatism/American College of Rheumatology) have been developed and partially validated.^13^, ^14^ The data were derived from a multi‐center trial culminating in the development of a probability score used to assess the probability of a patient having an inflammatory myopathy.^13^ Variables assessed include age at onset, muscle weakness, skin manifestations, dysphagia or esophageal dysmotility, laboratory measurements, and muscle biopsy features. Sensitivity and specificity were found to be improved over the previous Bohan and Peter's criteria.^7^, ^13^ The final score represents the probability of having an inflammatory myopathy. The recommendation is that a score of at least 5.5 (probability cutoff of 55%) is the suggested minimum for diagnosis. A score of equal to or greater than 7.5 suggests a probability of greater than 90%.^14^ A web‐based calculator has been developed (www.imm.ki.se/biostatistics/calculators/iim).^14^

Classically, diagnostic criteria for dermatomyositis must include at least one of the following (heliotrope rash, Gottron's papules, Gottron's sign) and must include any four of the following (proximal muscle weakness, elevated CPK or aldolase, muscle pain on grasping or spontaneous pain, EMG changes, positive anti‐Jo‐1 antibody test, non‐destructive arthritis or arthralgias, systemic inflammatory signs, or pathologic findings compatible with inflammatory myositis on biopsy and myopathic changes on EMG.^9^, ^12^ Characteristic findings on muscle biopsy include perifascicular atrophy/fibrosis. Predominant inflammatory infiltrate is perimysial, including CD4+, macrophages, and B cells.^15^ The differential diagnosis includes overlapping diseases such as SLE, Sjogrens, systemic sclerosis, HIV, seborrheic dermatitis, lichen planus, contact dermatitis, trichinosis, drug effects, and psoriasis among others.^12^

Complications of the disease can be fatal and include cardiopulmonary disease, renal failure, hematologic issues, and associated malignancies previously mentioned. Risk of developing complications can be modified by appropriate treatment. Pulmonary problems include interstitial lung disease with respiratory failure and opportunistic infection. Although acute kidney injury rarely occurs secondary to rhabdomyolysis associated with dermatomyositis, its presence during ICU admission is a predictor of short‐term mortality, as is lymphocytopenia.^16^ Thrombocytopenia has been reported but is very rare.^17^ Cardiac disease may occur and typically involves conduction abnormalities and arrhythmias. Congestive heart failure is rare, as is myocardial infarction and venous thromboembolism.^18^

First‐line treatment includes prednisone 0.5–1.5 mg/kg/day until the CPK normalizes, then tapered over 1 year.^12^ Methotrexate and Rituximab can be used for non‐responders.^1^ Janus kinase inhibitors and IVIG have been used for refractory cases as well.^5^ Treatment also includes physical therapy to improve physical endurance and help prevent the development of contractures.^12^

Given the association of dermatomyositis with cancer, aggressive screening is warranted in the work‐up.^1^, ^19^, ^20^ The most frequent cancers included breast, hematologic, colorectal, and prostate.^7^ Other studies have identified ovarian, nasopharyngeal, and others. Most occur within 1 year of diagnosing dermatomyositis.^2^ The exact mechanism of this relationship between cancer and dermatomyositis has yet to be elucidated. The term cancer‐associated myositis has been proposed as a paraneoplastic syndrome.^21^ High‐risk features associated with cancer‐associated myositis include old age, dermatomyositis, absence of interstitial lung disease, severe cutaneous necrotizing inflammation on biopsy, poor response to myositis treatment,^21^ and dysphagia.^22^ The most suggestive risk of cancer in myositis is associated with autoantibodies anti‐TIF1‐gamma and antinuclear matrix protein‐2.^22^ Myositis‐specific autoantibodies are observed in patients with idiopathic inflammatory myopathies exclusively, including dermatomyositis.^10^ However, only about 20% of dermatomyositis patients demonstrate a myositis‐specific autoantibody.^10^

Myositis‐specific autoantibodies (MSA) aid in subclassification of dermatomyositis as there are multiple phenotypes.^22^ This makes the diagnosis more difficult as each phenotype has unique features. Autoantibodies include Mi‐2, MDA‐t, NXP‐2, and TIF‐1ɤ (both associated with malignancy), SAE 1/2, ASAs (Jo‐1, PL‐12, PL‐7, EJ, OJ, KS, Zo, and Ha//YRS. The antisynthetase autoantibodies (ASAs) listed are associated with antisynthetase syndrome and have an increased risk of myositis.^22^ MSAs are thus helpful in diagnosing and subclassifying patients, thereby further directing patient treatment and monitoring for sequelae.

Cancer screening is based on risk: basic, enhanced, or comprehensive.^23^ Basic occurs at diagnosis and every 3 years. It includes a history/physical, basic laboratories, colonoscopy, mammography, and screening for cervical cancer and prostate cancer. Enhanced screening includes basic plus one or more of the following: computerized tomography of the chest/abdomen/pelvis, tumor markers, pelvic ultrasound in females, or testicular ultrasound in males. Comprehensive screening includes basic or enhanced plus consideration of positron emission tomography scanning with computerized tomography of the chest/abdomen/pelvis.^23^

Dermatomyositis is a rare disorder, having a broad range of clinical presentations, with significant potential for morbidity and mortality. Thus, potential for delayed diagnosis is a concern. Given the propensity for progression with increasing morbidity as well as the association with internal malignancy and other complications, a timely and thorough work‐up is required. Dermatomyositis should remain on the differential for patients presenting with diffuse muscle weakness and the presence of a rash, although a rash may be the sole presenting symptom. Although confirmation of the diagnosis will require testing not available in the ED, treatment should begin as soon as there is high suspicion of the illness. Severe cases may require hospital admission.

## CONFLICT OF INTEREST

None declared.

## AUTHOR CONTRIBUTIONS

All authors contributed to the manuscript.

## ETHICAL APPROVAL

I certify that this material has not been published elsewhere, either in whole or part, and is not under consideration for publication in any other journal. I have personally and actively been involved in substantive work leading to the revised manuscript and will hold themselves jointly and individually responsible for its content.

## Data Availability

Data sharing is not applicable to this article as no new data were created or analyzed in this study.
